# Effect of domperidone, ondansetron, olanzapine-containing antiemetic regimen on QT_C_ interval in patients with malignancy: a prospective, observational, single-group, assessor-blinded study

**DOI:** 10.1038/s41598-020-79380-1

**Published:** 2021-01-11

**Authors:** Ashwin Kamath, K. Maneesh Rai, R. Shreyas, PU Prakash Saxena, Sourjya Banerjee

**Affiliations:** 1 Department of Pharmacology, Kasturba Medical College, Mangalore, Manipal Academy of Higher Education, Manipal, Karnataka 575001 India; 2 Department of Cardiology, Kasturba Medical College, Mangalore, Manipal Academy of Higher Education, Manipal, Karnataka 575001 India; 3 Department of Radiation Oncology, Kasturba Medical College, Mangalore, Manipal Academy of Higher Education, Manipal, Karnataka 575001 India

**Keywords:** Cardiology, Oncology

## Abstract

Domperidone, ondansetron and olanzapine can prolong the QT interval. The clinical use of combinations of these drugs is not uncommon. Our study aimed to determine the presence of any QTc prolonging effect of the combination when used as antiemetic in patients receiving cancer chemotherapy. We carried out a prospective, observational study of patients with malignancy who were to receive domperidone, ondansetron and olanzapine-containing antiemetic regimen. Electrocardiograms were recorded before and during the administration of antiemetics, for three consecutive days. A blinded assessor determined the QTc interval using Bazett and Fridericia formulae. Thirty-six patients completed the study; 23 (63.9%) were females. There was a statistically significant change in QTc with time (Fridericia, χ^2^(4) = 15.629, *p* = 0.004; Bazett, χ^2^(4) = 15.910, *p* = 0.003); QTc on Day 1 was more than that during baseline (*p* < 0.001); these differences were significant in females (Fridericia, χ^2^(4) = 13.753, *p* = 0.008; Bazett, χ^2^ (4) = 13.278, *p* = 0.010) but not in males (Fridericia, χ^2^ (4) = 4.419, *p* = 0.352; Bazett, χ^2^(4) = 4.280, *p* = 0.369). Two female patients had an absolute QTc prolongation (Bazett correction) of > 500 ms. However, no clinically significant adverse events occurred. The findings show that QTc prolongation is a concern with olanzapine alone and in combination with domperidone and ondansetron, and needs to be investigated further.

## Introduction

The use of antiemetics is an integral part of cancer chemotherapy and radiotherapy. Not only do they significantly reduce the incidence of nausea and vomiting and, thereby, improve the quality of life but also enable the use of high-dose chemotherapy resulting in better treatment response^1^. Drugs such as dopamine D2 receptor antagonists, 5-HT_3_ antagonists, glucocorticoids and neurokinin receptor antagonists are used in various combinations, depending on the emetogenic risk of chemotherapy or the extent of irradiation. The combination of glucocorticoids with 5-HT_3_ antagonists is synergistic and is a commonly used combination to manage the acute phase of cancer-induced vomiting. Olanzapine and aprepitant can be added to this combination for highly-emetogenic chemotherapy^1^. Olanzapine is a potentially safer and cost-effective alternative to aprepitant to prevent nausea and vomiting^2^. Domperidone is a commonly used drug for the delayed phase of cancer chemotherapy-induced nausea and vomiting^3^. A combination of olanzapine and dexamethasone to prevent acute phase nausea and vomiting and short-term ondansetron and domperidone for the delayed phase is a cost-effective treatment approach compared with aprepitant-containing regimens in resource-poor settings^2^. An open-label interventional study comparing the combination of domperidone with ondansetron to ondansetron alone found that the former produced a more pronounced antiemetic effect without any significant adverse effects in breast cancer patients on moderately emetogenic chemotherapy^4^. The addition of domperidone to other antiemetic drug regimens in non-responsive cases and those receiving total body irradiation has also been described in some of the treatment guidelines^5,6^. However, one of the important drawbacks of domperidone is its tendency to cause QT prolongation, particularly in the presence of other drugs in the antiemetic regimen with QT-prolonging potential^7^. The extent to which such interactions are clinically relevant to the short-term use of domperidone at therapeutic doses is unclear. Studies of domperidone in healthy volunteers, as well as patients, have not shown any significant QT-prolonging effects in the recommended doses^8,9^. During a recent study to identify potentially interacting drug combinations using a proprietary drug interaction detection software in patients undergoing chemoradiation, a downgrading of the severity of drug-drug interaction for the combination of domperidone with ondansetron in the software database was noted^10^; the DDI was downgraded from category X, requiring the drug combination to be avoided, to category D, requiring modification of therapy. The combination of domperidone, ondansetron, and olanzapine is used at the study site in patients being treated with highly emetogenic chemotherapy and high-dose radiation. Considering the high risk of QT prolongation with the use of domperidone and moderate risk with ondansetron^11,12^ and olanzapine^13–15^, we aimed to determine the presence of any QT-prolonging effect of the combination (domperidone, ondansetron and olanzapine) when used for prevention of nausea and vomiting in patients on cancer chemotherapy.

## Methods

A prospective, observational study was carried out at the Radiation Oncology department of Kasturba Medical College Hospital, Mangalore, a tertiary care teaching hospital in South India. Patients with malignancy aged 18 − 80 years, of either gender, Eastern Cooperative Oncology Group status ≤ 2, who were scheduled to receive domperidone, ondansetron and olanzapine-containing antiemetic regimen, and willing to provide written informed consent were included in the study. Patients with a baseline QTc interval > 450 ms, history of cardiac arrhythmias, history of additional risk factors for Torsades de Pointes (e.g., heart failure, hypokalemia, family history of long QT syndrome), use of concomitant medications that prolong the QT/QTc (other than the study drugs), unable to complete the study as per the opinion of the investigators were excluded from the study.

The study was initiated after receiving approval from Kasturba Medical College Institutional Ethics Committee (IEC KMC MLR 08–18/165) and registration of the study protocol in the Clinical Trial Registry of India (CTRI/2018/09/015676). The study was conducted in accordance with the Ethical Guidelines for Biomedical Research on Human Subjects (Indian Council of Medical Research) and the Declaration of Helsinki. After obtaining written informed consent from the participants, serial electrocardiograms (ECGs) were recorded at specific time points over three days; the antiemetic drugs used, their dose, and frequency of administration are shown in Table 1.

**Table 1:** The antiemetic regimen used at the study site in patients with malignancy treated with highly emetogenic chemotherapy and/or high-dose radiation.

Antiemetic regimen	Activity	Number of ECGs recorded per day
**Day 0**		
Baseline	Two ECGs recorded at least 10 min apart within 24 h before the administration of the antiemetic on Day 1	2
**Day 1**		
Injection Palonosetron 0.25 mg OD Tablet Olanzapine 10 mg OD	One ECG recorded 6 h (± 30 min) after the antiemetic dose on Day 1	1
**Day 2**		
Tablet Olanzapine 10 mg OD Tablet Pantoprazole 40 mg OD Tablet Domperidone 10 mg BID Tablet Ondansetron 8 mg TID	1 h (± 15 min; Day 2_1) and 2 h (± 15 min; Day 2_2) after the first antiemetic dose on Day 2	2
**Day 3**		
Tablet Olanzapine 10 mg OD Tablet Pantoprazole 40 mg OD Tablet Domperidone 10 mg BID Tablet Ondansetron 8 mg TID	4 h (± 30 min) after the first antiemetic dose on Day 3	1

ECG, electrocardiogram; OD, once a day; BID, twice a day; TID, thrice a day.

The ECGs were recorded at the specified time points using Cardiart 9108 12-channel ECG machine (BPL Medical Technologies, India) with a paper speed of 25 mm/s, amplitude 1 mV/10 mm, high pass filter at 0.5 Hz and low pass filter at 40 Hz, with the time of administration of the first antiemetic on Day 1 determining the time to record the subsequent ECGs. The ECG recordings of all the patients were assessed by a cardiologist to manually determine the QT interval corrected for heart rate, who was blinded to the patient identity, the day and time point of the recording of the ECGs; the end of T-wave was determined using the tangent method. The QT correction was performed using Bazett and Fridericia formulae^8^. Intra-reader variability was determined by reassessment of the QTc for 10% of the recorded ECGs by the same evaluator on a different occasion.

### Statistical analysis

The heart rate, QTc interval and change from baseline in heart rate and QTc for each time point of measurement are summarized using descriptive statistics. Intraclass correlation coefficient was measured to determine the intra-reader variability. The number of ECGs with an absolute QTc interval > 450, > 480 and > 500 ms was determined; also, an increase in QTc values from baseline by > 30 and > 60 ms was noted^8^; Chi-square test was used for group comparisons. Patients ≥ 50 years of age were considered elderly; this arbitrary cut-off limit was based on the age distribution of the study population. Since the data was not normally distributed (as determined using the Shapiro–Wilk test; *p* < 0.05), nonparametric repeated-measures analysis of variance (Freidman’s test) was used to determine the presence of any significant difference in the QTc interval before and after administration of the drugs. If a significant difference in the QTc interval was seen with time, Wilcoxon signed-rank test was used to identify the time points with a significant difference, using Bonferroni correction to adjust for multiple comparisons. A *p* value < 0.05 was considered statistically significant.

The sample size was calculated based on the assumption that, for a sample size of 36 patients, with a probability of 80% and a two-sided significance level of 0.05 and estimated standard deviation in the population of 10 ms^9^, the minimum effect size that can be detected is 5 ms (calculated using University of California San Francisco Online Sample Size Calculator). The sample size was determined to be 40 considering a screen failure rate of 10%.

## Results

Forty patients were screened for the study. Four patients were excluded due to prolonged QTc (> 450 ms) at baseline. Of the 36 patients who completed the study, 23 (63.9%) were females; the median age of males was 55 years (interquartile range [IQR], 48–60) and females 45 (41–58) years (*p* = 0.202). There was no significant difference in gender distribution among young adults and the elderly (χ^2^(1) = 1.084, *p* = 0.298); 27.78% (10/36) had breast malignancy, 19.44% (7/36) oropharyngeal, 16.67% (6/36) ovarian and 11.11% (4/36) oesophageal malignancy. The intraclass correlation coefficient for QTc assessment using Fridericia formula was 0.928 (95% confidence interval, 0.842–0.968), suggesting good to excellent intra-reader measurement reliability.

**Figure 1:**
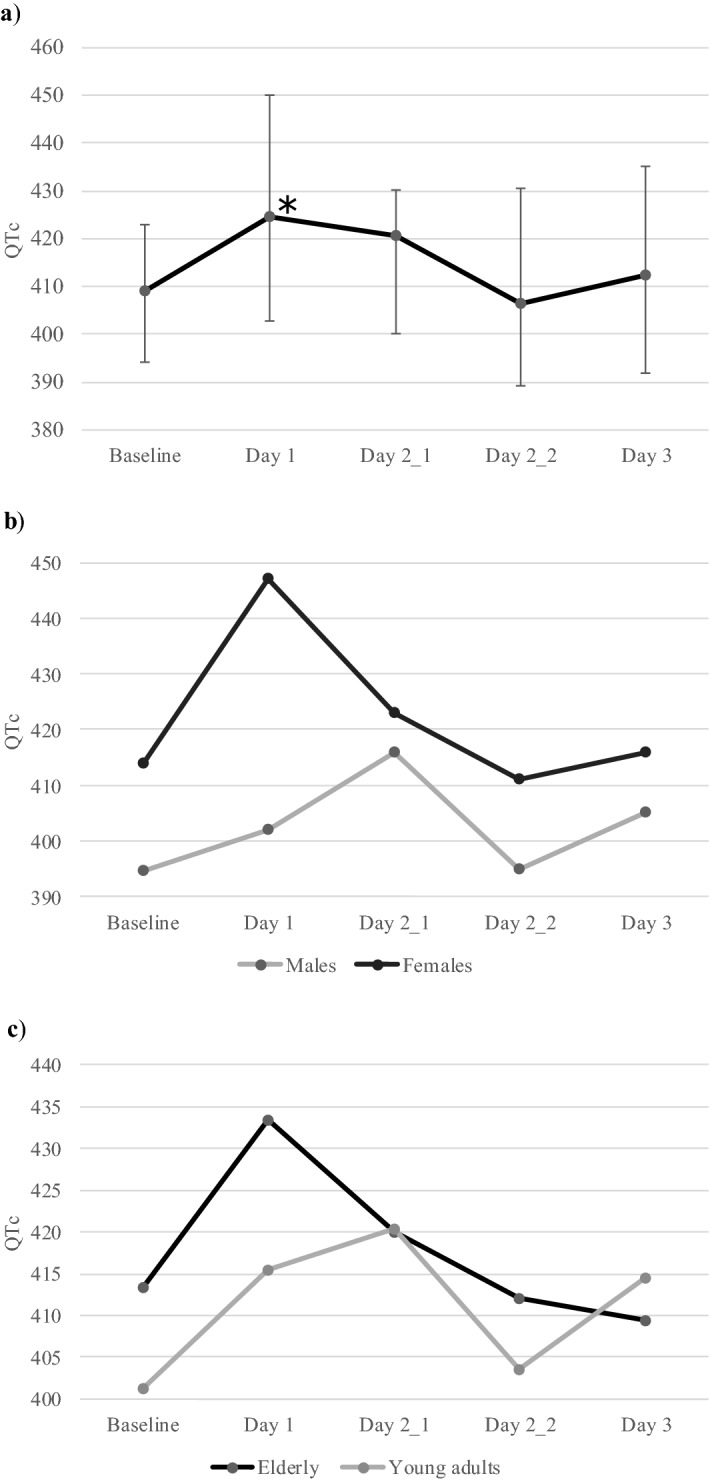
Change in QTc (corrected using Bazett formula) with time. (**a**) The median QTc values with interquartile range (vertical bars) at different study time points in patients with malignancy receiving antiemetics with QT-prolonging potential are shown. *p < 0.01 for comparison with baseline, second timepoint on day 2 and day 3. (**b**) The median QTc values in male and female patients at different study time points are shown. The variation in QTc with time was statistically significant in females (*p* < 0.05). (**c**) The median QTc values in young adults and elderly patients (≥ 50 years) at different study time points are shown. The variation in QTc with time was statistically significant in the elderly (*p* < 0.05). Day 2_1, first time point on Day 2; Day 2_2, second time point on Day 2.

We compared the median QTc values at different time points with that at baseline (Fig. 1, Table 2). There was a statistically significant change in the QTc with time (Fridericia, χ^2^(4) = 15.629, *p* = 0.004; Bazett, χ^2^(4) = 15.910, *p* = 0.003). Post hoc analysis with Wilcoxon signed-rank tests was conducted with a Bonferroni correction applied, resulting in a significance level set at *p* < 0.005. Compared with baseline QTc (median [IQR], 386.0 ms [373.3–399.5]), assessed using Fridericia formula, QTc on Day 1 (399.0 ms [388.3–420.3]; Z =  − 3.784, *p* < 0.0001) and first time point of Day 2 (399.0 ms [380.0–421.8]; Z =  − 3.080, *p* < 0.002) was significantly prolonged. Bazett’s correction showed a statistically significant increase in QTc on Day 1 (median [IQR], 424.5 ms [402.8–450.0]) compared with baseline (409.3 ms [394.1–423.1]; Z =  − 3.865, *p* < 0.0001), second time point of Day 2 (Day 2_2; 406.5 ms [389.3–430.5]; Z =  − 3.088, *p* = 0.002) and Day 3 (412.5 ms [392.0–435.3]; Z =  − 3.172, *p* = 0.002). On analysis based on gender, these differences were significant in females (Fridericia, χ^2^(4) = 13.753, *p* = 0.008; Bazett, χ^2^(4) = 13.278, *p* = 0.010) but not in males (Fridericia, χ^2^(4) = 4.419, *p* = 0.352; Bazett, χ^2^(4) = 4.280, *p* = 0.369).

**Table 2:** Change in QTc (corrected using Fridericia formula) with time in patients with malignancy receiving antiemetic drugs with QT-prolonging potential.

Study time point	QTc in milliseconds, Median (25th to 75th percentile)				
	Overall, N = 36	Males, N = 13	Females, N = 23	Young, N = 18	Elderly, N = 18
Baseline	386 (373–400)	379 (365–391)	388 (379–402)	385 (374–405)	387 (367–399)
Day 1	399 (388–420)	391 (380–399)	417 (395–433)	405 (388–434)	397 (387–419)
Day 2_1	399 (380–422)	405 (365–431)	398 (382–421)	400 (382–419)	397 (371–425)
Day 2_2	394 (379–410)	395 (375–414)	393 (379–410)	396 (379–412)	393 (375–410)
Day 3	396 (374–414)	379 (340–410)	403 (381–415)	395 (373–410)	404 (368–426)

QTc values have been rounded off to whole number.

Table 3 shows the number of patients with at least one of the electrocardiograms (ECGs) with QTc > 450 ms, > 480 ms or > 500 ms, after drug administration, based on gender. There was no statistically significant gender difference with regard to the occurrence of QTc prolongation (Fridericia, χ^2^(1) = 3.282, *p* = 0.070; Bazett, χ^2^(1) = 0.994, *p* = 0.319). However, two female patients had an absolute QTc prolongation (Bazett’s correction) of > 500 ms, which is considered a clinically significant threshold for the risk of serious arrhythmias.

**Table 3:** Absolute increase in QTc interval in patients with malignancy receiving antiemetic drugs with QT-prolonging potential.

Variable	QTc prolongation (N)					
	> 450–480 ms		> 480–500 ms		> 500 ms	
	Fridericia	Bazett	Fridericia	Bazett	Fridericia	Bazett
**Gender**						
Male (N = 13)	0	4	0	0	0	0
Female (N = 23)	2	8	2	1	1	2
**Study time point** (N = 36)						
Day 1	2	7	0	0	0	0
Day 2_1	1	4	2	1	0	1
Day 2_2	0	0	0	0	1	1
Day 3	1	4	0	0	0	0

Day 2_1, first time point on Day 2; Day 2_2, second time point on Day 2.

Table 4 shows the number of patients with at least one of the ECGs with ∆QTc > 30 ms or > 60 ms, compared with the baseline value. Overall, no statistically significant gender difference was seen (Fridericia, χ^2^(1) = 0.890, *p* = 0.346; Bazett, χ^2^(1) = 0.358, *p* = 0.549), although, more female patients had QTc prolongation of more than 60 ms compared with males (Bazett’s correction, 13.04% versus 7.69%, respectively).

**Table 4:** QTc interval increase from baseline (∆QTc) in patients with malignancy receiving antiemetic drugs with QT-prolonging potential.

Variable	∆QTc (N)			
	> 30 ms		> 60 ms	
	Fridericia	Bazett	Fridericia	Bazett
**Gender**				
Male (N = 13)	7	5	0	1
Female (N = 23)	11	10	5	3
**Study time point (N = 36)**				
Day 1	10	10	3	1
Day 2_1	8	3	2	3
Day 2_2	7	6	1	1
Day 3	6	6	0	0

Day 2_1, first time point on Day 2; Day 2_2, second time point on Day 2.

**Figure 2:**
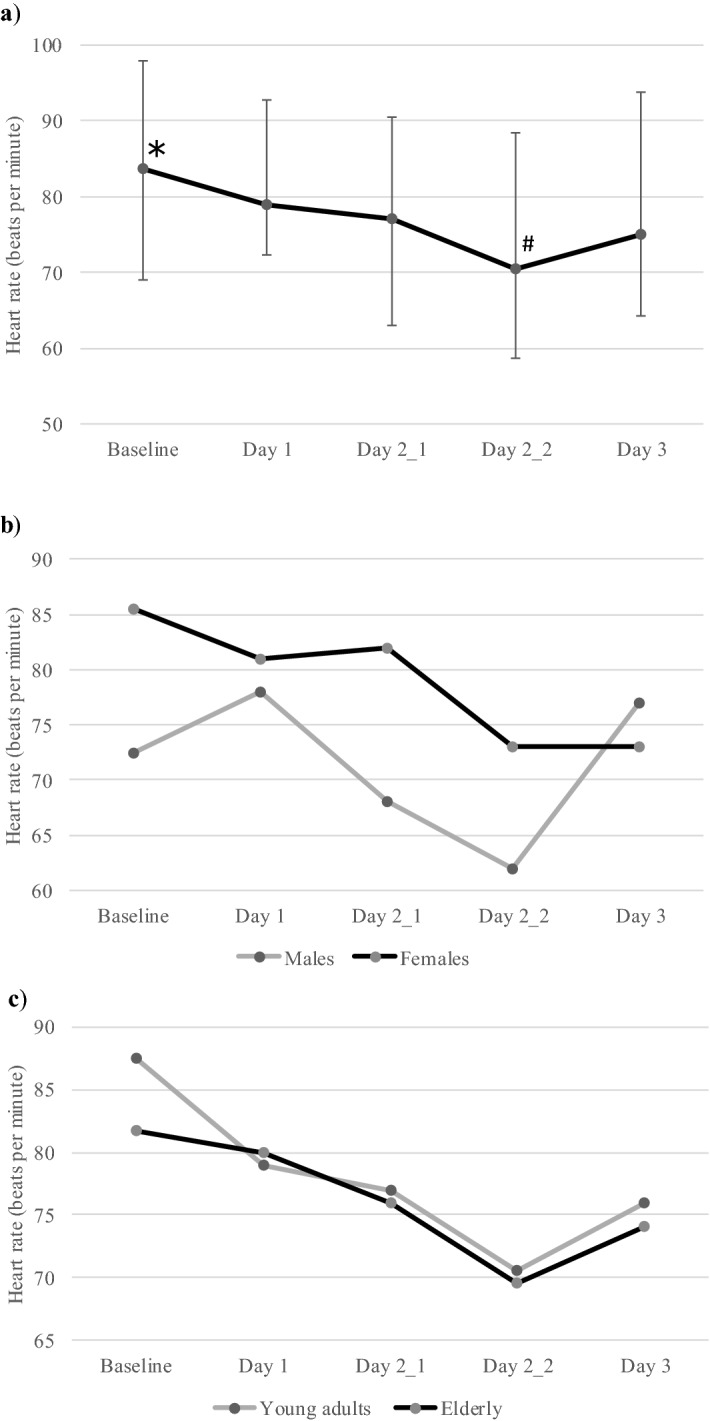
Change in heart rate with time. (**a**) The median heart rate and interquartile range (vertical bars) at different study time points in patients with malignancy receiving antiemetics with QT-prolonging potential are shown. *p < 0.01 for comparison with first and second time point on Day 2; # *p* < 0.01 for comparison with Day 1 and first timepoint of Day 2. (**b**) The median heart rate in male and female patients at different study time points are shown. The variation in heart rate with time was statistically significant in females (*p* < 0.001). (**c**) The median heart rate in young adults and elderly patients (≥ 50 years) at different study time points are shown. The variation in heart rate with time was statistically significant in both the groups (*p* < 0.05). Day 2_1, first time point on Day 2; Day 2_2, second time point on Day 2.

We compared the median heart rate (HR) at different time points with that at baseline (Fig. 2). There was a statistically significant change in the HR with time (χ^2^(4) = 29.787, *p* < 0.001). Post hoc analysis was conducted with a Bonferroni correction applied, resulting in a significance level set at *p* < 0.005. There was a statistically significant decrease in HR on first (Day 2_1; 77 beats per minute [bpm; 63–91]; Z =  − 3.499, *p* < 0.001) and second time point of Day 2 (Day 2_2; 71 bpm [59–89]); Z =  − 4.403, *p* < 0.001) compared with baseline (84 bpm [69–98]); on Day 2_2 (71 bpm [59–89]) compared with Day 1 (79 bpm [72–93]; Z =  − 3.449, *p* = 0.001) and Day 2_1 (77 bpm [63–91]; Z =  − 3.309, *p* = 0.001). On analysis based on gender, these differences were significant in females (χ^2^(4) = 27.483, *p* < 0.001) but not in males (χ^2^(4) = 9.150, *p* = 0.057).

A similar trend of a significant decrease in HR with time was seen in young adults (χ2(4) = 19.921, *p* = 0.001) as well as elderly (χ2(4) = 12.596, *p* < 0.013). However, a significant change in the QTc with time was seen in the elderly (Fridericia, χ^2^(4) = 7.796, *p* = 0.099; Bazett, χ^2^(4) = 11.003, *p* = 0.027) but not young adults (Fridericia, χ^2^(4) = 8.858, *p* = 0.065; Bazett, χ^2^(4) = 5.732, *p* = 0.220) only when Bazett formula was applied.

## Discussion

We studied the effects of the combination of domperidone, ondansetron and olanzapine, administered in patients receiving highly emetogenic chemotherapy and/or radiotherapy, on the QTc interval. Although palonosetron was also a part of the antiemetic regimen, it does not have any significant QT-prolonging potential, and in particular, has no pharmacologic interaction with domperidone^13^. Our study showed that there was a significant prolongation in the QTc following administration of olanzapine on Day 1; this was significantly more than the QTc at baseline. Administration of domperidone and ondansetron on Day 2, along with olanzapine, did not show further significant prolongation of QTc. However, elevations in absolute QTc > 480 ms occurred on Day 2, suggesting that the combination may produce clinically significant QTc prolongations in some individuals, and hence, is better avoided. In general, the maximum number of QTc prolongations occurred on Day 1, whereas more severe QTc prolongations occurred on Day 2. No participants, however, developed clinically identifiable arrhythmias. We used two formulae for QT correction; Bazett formula is widely used in clinical settings, and thorough QT/QTc study guidelines recommend Fridericia formula^8^. Bazett formula has been shown to overestimate the QTc prolongation and performs relatively poorly at higher heart rates^16,17^. While this was true even in the current study, there was an overall agreement in the study findings irrespective of the correction formula used. Analysis of the heart rate shows a fall in the heart rate compared with baseline and Day 1. However, the changes in the heart rate were not clinically significant because the interquartile ranges did not exceed the lower limit of 50 or upper limit of 100 beats/minute.

Although the antiemetic regimen used in our study may not be the standard practice at other sites, the findings provide clinically significant information regarding the safety of drug combinations with potential QT-prolonging effects. QTc changes with time were significant in females and elderly in our study; this is in agreement with the findings of a retrospective ECG review of hospitalized psychiatry patients on psychotropic drugs^18^. The gender difference is likely to be sex hormone-related^19^ whereas the age difference is likely to be due to the effect of ageing on the myocardium and autonomic tone^20^; interestingly, the latter study showed that in elderly, the QTc prolongation is more severe in males compared with females.

Our study shows that olanzapine has a QT-prolonging effect, particularly on the day of initiation. Studies, mainly case reports, have reported an increased risk of QT prolongation with atypical antipsychotics^15^; an increased risk of sudden cardiac death has also been shown for olanzapine^14,21^. However, the association between QT prolongation due to atypical antipsychotics and cardiac death has not been well established^22^. In fact, a study showed that despite the prolongation in QT interval with atypical antipsychotics, there was no increased risk of torsades de pointes^23^. Nonetheless, olanzapine has a low risk of QT prolongation^24^, and the risk increases with dose^14^. The dose used in our study was moderate, which has been shown to significantly increase the risk of cardiac death^14^. Guidelines recommend decreasing the dose or switching to safer alternatives when QTc is > 440 ms in males and > 470 ms in females, and stopping the drug when > 500 ms^24^.

Despite being a drug with a high risk of QT prolongation, domperidone did not produce a statistically significant increase in the QTc interval; in fact, using Bazett formula, the QTc on day 1 was significantly higher than on Day 2 when domperidone was introduced along with ondansetron. However, when the absolute and delta values are seen, severe increases in QTc interval, which are clinically important, occurred on Day 2. Like in the case of olanzapine, there are several case reports linking domperidone with life-threatening QT prolongation^7^. However, most of these have been with intravenous domperidone. A case–control study also showed an increased risk of sudden cardiac death^25^. A systematic review and meta-analysis also showed an increased risk of ventricular arrhythmias and sudden cardiac death by up to 70%^26^. However, some of the recent studies have failed to show an increased risk with domperidone, both at high antiemetic doses^27^ and low doses < 30 mg/day^28^. A similar risk profile is also present for ondansetron, where, despite its QT-prolonging potential, clinically significant arrhythmias are uncommon^12^. The lack of evidence of significant QT-prolonging risk in recent studies resulted in the downgrading of the risk category for the interaction between domperidone and ondansetron^10^.

Considering that prescription of a combination of QT-prolonging drugs, particularly those containing domperidone, is not uncommon^10,29^, the likely impact of such combinations on the risk of QT prolongation is important. An evaluation of such combinations in psychiatry patients showed that the risk class is more important rather than the number of coadministered QT-prolonging drugs^30^. The effect of the drugs did not seem to persist on Day 3, both in terms of change with time and absolute/delta values. One probable reason might be the different time points following the drug administration at which the ECGs were obtained. We used different time points to coincide with the peak drug concentrations (Day 1, peak concentration of olanzapine; Day 2_1, peak concentration of domperidone) as well as the non-peak effects (Day 2_2 and Day 3). It is also possible that the peak effect of olanzapine on QT interval occurs initially, coinciding with the autonomic effects of the drug, although there is no evidence to substantiate this. Moreover, contrary to the expected effect of the drug on heart rate^31^, a decrease in the heart rate was seen in our study. Notwithstanding the above observations, our study shows that QTc prolongation is a concern with olanzapine alone and in combination with domperidone and ondansetron. Whether this QT prolongation is adequate to cause clinical events is unclear, which is in line with the findings of earlier studies.

Our study has limitations. It was a single-centre study with small sample size. There was no control group; hence, it is not possible to definitely attribute the prolonged QTc observed in the study to be due to the study drugs. However, we intended to study the effects of the antiemetics in a real-world setting, and hence, including a control for each antiemetic studied was not feasible. Although the assessor (cardiologist) was blinded, having two persons read the ECG would have better eliminated reading errors. The time points were based on the time of administration of the first dose of the antiemetic combination; hence, the ECGs were recorded at different times of the day in the study participants. Although we have described significant differences in the measured parameters based on patient age and gender, the study was not primarily designed to evaluate these differences; the findings, therefore, need to be explored further in well-designed studies. We excluded patients receiving other QT-prolonging drugs (other than the study drugs); hence, the generalizability of our study findings is limited.

To conclude, our study showed that the combination of domperidone, ondansetron and olanzapine could cause potentially clinically significant QTc prolongation; caution needs to be exercised, particularly in females and elderly patients. Olanzapine alone can cause significant QTc prolongation. Targeted safety assessments in patients receiving these drugs with QT-prolonging potential in real-world settings are necessary to determine whether the QT-prolonging effects do translate into adverse clinical outcomes.

## Data Availability

The datasets generated during and/or analysed during the current study are available from the corresponding author on reasonable request.

## References

[1] Hesketh PJ (2017). Antiemetics: American society of clinical oncology clinical practice guideline update. J. Clin. Oncol..

[2] Babu G (2016). The efficacy, safety, and cost benefit of olanzapine versus aprepitant in highly emetogenic chemotherapy: a pilot study from South India. Chemother. Res. Pract..

[3] Powell, R. West Midlands Expert Advisory Group for Systemic Anti-cancer Therapy (SACT). Guideline for the use of Anti-emetics in Adults Receiving Anti-Cancer Drug Therapy. NHS, UK. Available at https://www.england.nhs.uk/mids-east/wp-content/uploads/sites/7/2018/04/use-of-anti-emetics-in-adults-receiving-anti-cancer-drug-therapy.pdf (2017).

[4] Gupta KD (2007). Effects of ondansetron alone and in combination with domperidone in the prevention of chemotherapy-induced nausea and vomiting in breast cancer patients. Bangladesh J. Pharmacol..

[5] Antiemetic Guidelines for Chemotherapy and Radiotherapy. Canterbury District Health Board, New Zealand. Available at https://www.cdhb.health.nz/Hospitals-Services/Cancer-Blood-Services/Cytotoxic-Biotherapy/Documents/Oncology%20Antiemetic%20Policy.pdf (2012).

[6] Antiemetic Guidelines for Adult Patients Receiving Chemotherapy and Radiotherapy. NHS, UK. Available at http://www.londoncancer.org/media/65597/antiemetic-guidelines-november-2010.pdf (2010).

[7] Rossi M, Giorgi G (2010). Domperidone and long QT syndrome. Curr. Drug Saf..

[8] US Food and Drug Administration. ICH guidance for industry: E14 Clinical Evaluation of QT/QTc Interval Prolongation and Proarrhythmic Potential for Non-Antiarrhythmic Drugs. Available at http://www.fda.gov/downloads/Drugs/GuidanceComplianceRegulatoryInformation/Guidances/UCM073153.pdf (2005). 16237860

[9] Biewenga J (2015). Absence of QTc prolongation with domperidone: a randomized, double-blind, placebo- and positive-controlled thorough QT/QTc study in healthy volunteers. Clin. Pharmacol. Drug Dev..

[10] Daggupati SJV, Saxena PUP, Kamath A, Chowta MN (2019). Drug-drug interactions in patients undergoing chemoradiotherapy and the impact of an expert team intervention. Int. J. Clin. Pharm..

[11] Hafermann MJ, Namdar R, Seibold GE, Page RL (2011). Effect of intravenous ondansetron on QT interval prolongation in patients with cardiovascular disease and additional risk factors for torsades: a prospective, observational study. Drug Healthc. Patient Saf..

[12] Moffett PM, Cartwright L, Grossart EA, O'Keefe D, Kang CS (2016). Intravenous ondansetron and the QT interval in adult emergency department patients: an observational study. Acad. Emerg. Med..

[13] Lexicomp Online, Lexicomp Drug Interactions, Hudson, Ohio: UpToDate, Inc. Available from https://www.uptodate.com/drug-interactions/?source=responsive_home#di-druglist (2020).

[14] Ray WA, Chung CP, Murray KT, Hall K, Stein CM (2009). Atypical antipsychotic drugs and the risk of sudden cardiac death. N. Engl. J. Med..

[15] Vieweg WVR (2003). New generation antipsychotic drugs and QTc interval prolongation. Prim. Care Companion J. Clin. Psychiatry..

[16] Strohmer B, Schernthanere C, Paulweber B, Pichler M (2007). Gender-specific comparison of five QT correction formulae in middle-aged participants in an atherosclerosis prevention program. Med. Sci. Monit..

[17] Vandenberk B (2016). Which QT correction formulae to use for QT monitoring?. J. Am. Heart Assoc..

[18] Ansermot N (2019). Prevalence of ECG abnormalities and risk factors for QTc interval prolongation in hospitalized psychiatric patients. Ther. Adv. Psychopharmacol..

[19] Vink AS, Clur SB, Wilde AAM, Blom NA (2018). Effect of age and gender on the QTc-interval in healthy individuals and patients with long-QT syndrome. Trends Cardiovasc. Med..

[20] Rabkin SW, Cheng XJ, Thompson DJ (2016). Detailed analysis of the impact of age on the QT interval. J. Geriatr. Cardiol..

[21] Jones ME (2013). Risk of mortality (including sudden cardiac death) and major cardiovascular events in users of olanzapine and other antipsychotics: a study with the general practice research database. Cardiovasc. Psychiatry Neurol..

[22] Czekalla J, Beasley CM, Dellva MA, Berg PH, Grundy S (2001). Analysis of the QTc interval during olanzapine treatment of patients with schizophrenia and related psychosis. J. Clin. Psychiatry..

[23] Hasnain M, Vieweg WV (2014). QTc interval prolongation and torsade de pointes associated with second-generation antipsychotics and antidepressants: a comprehensive review. CNS Drugs.

[24] Lambiase PD (2019). British heart rhythm society clinical practice guidelines on the management of patients developing QT prolongation on Antipsychotic Medication. Arrhythm Electrophysiol. Rev..

[25] van Noord C, Dieleman JP, van Herpen G, Verhamme K, Sturkenboom MC (2010). Domperidone and ventricular arrhythmia or sudden cardiac death: a population-based case-control study in the Netherlands. Drug Saf..

[26] Leelakanok N, Holcombe A, Schweizer ML (2016). Domperidone and risk of ventricular arrhythmia and cardiac death: a systematic review and meta-analysis. Clin. Drug Investig..

[27] Ortiz A (2015). Cardiovascular safety profile and clinical experience with high-dose domperidone therapy for nausea and vomiting. Am. J. Med. Sci..

[28] Bor S, Demir M, Ozdemir O, Yuksel K (2018). A meta-analysis on the cardiac safety profile of domperidone compared to metoclopramide. United European Gastroenterol. J..

[29] Balodiya S, Kamath A (2019). Evaluation of hospital discharge prescriptions in the elderly and younger adults using the medication regimen complexity index. Curr. Drug Saf..

[30] Meid AD (2017). Combinations of QTc-prolonging drugs: towards disentangling pharmacokinetic and pharmacodynamic effects in their potentially additive nature. Ther. Adv. Psychopharmacol..

[31] Tajiri M, Suzuki Y, Sugai T, Tsuneyama N, Someya T (2018). Effects of olanzapine on resting heart rate in Japanese patients with schizophrenia. PLoS ONE.

